# ADM3, TFF3 and LGALS3 are discriminative molecular markers in fine-needle aspiration biopsies of benign and malignant thyroid tumours

**DOI:** 10.1038/bjc.2011.578

**Published:** 2012-01-05

**Authors:** S Karger, K Krause, M Gutknecht, K Schierle, D Graf, F Steinert, H Dralle, D Führer

**Affiliations:** 1 Department of Internal Medicine, Neurology and Dermatology, Clinic for Endocrinology and Nephrology, University of Leipzig, Liebigstr. 18, 04103 Leipzig, Germany; 2 Department of Diagnostic Medicine, Institute of Pathology, University of Leipzig, Liebigstr. 26, 04103 Leipzig, Germany; 3 Private Practice of Endocrinology and Nuclear Medicine, Auf dem Meere 9, 21335 Lüneburg, Germany; 4 Department of Surgery, Helios Clinic Schkeuditz, Leipziger Straße 45, 04435 Schkeuditz, Germany; 5 Department of Surgery, University of Halle, Ernst-Grube-Straße 40, 06120 Halle (Saale), Germany; 6 Department of Medicine, Clinic of Endocrinology, University of Essen, Hufelandstr. 55, 45147 Essen, Germany

**Keywords:** thyroid tumours, molecular markers, fine-needle aspiration biopsy

## Abstract

**Background:**

Previously, we reported a six-marker gene set, which allowed a molecular discrimination of benign and malignant thyroid tumours. Now, we evaluated these markers in fine-needle aspiration biopsies (FNAB) in a prospective, independent series of thyroid tumours with proven histological outcome.

**Methods:**

Quantitative RT–PCR was performed (*ADM3*, *HGD1*, *LGALS3*, *PLAB*, *TFF3*, *TG*) in the needle wash-out of 156 FNAB of follicular adenoma (FA), adenomatous nodules, follicular and papillary thyroid cancers (TC) and normal thyroid tissues (NT).

**Results:**

Significant expression differences were found for *TFF3*, *HGD1*, *ADM3* and *LGALS3* in FNAB of TC compared with benign thyroid nodules and NT. Using two-marker gene sets, a specific FNAB distinction of benign and malignant tumours was achieved with negative predictive values (NPV) up to 0.78 and positive predictive values (PPV) up to 0.84. Two FNAB marker gene combinations (*ADM3/TFF3*; *ADM3/ACTB*) allowed the distinction of FA and malignant follicular neoplasia with NPV up to 0.94 and PPV up to 0.86.

**Conclusion:**

We demonstrate that molecular FNAB diagnosis of benign and malignant thyroid tumours including follicular neoplasia is possible with recently identified marker gene combinations. We propose multi-centre FNAB studies on these markers to bring this promising diagnostic tool closer to clinical practice.

Nodular thyroid disease is one of the most frequent endocrine disorders with an incidence of up to 50%, depending on the diagnostic criteria, in patients beyond the fifth decade of life (Gharib, 2004). Fine-needle aspiration biopsy (FNAB) is widely advocated as the most sensitive and specific tool for preoperative identification of rare thyroid malignancy. However, there are diagnostic uncertainties and technical shortcomings of FNAB, such as operator dependency and a lack of diagnostic markers in the case of indeterminate and/or suspicious FNAB (Baloch *et al*, 1998; Hegedüs *et al*, 2003; Sclabas *et al*, 2003). Molecular diagnosis might contribute to an improved assessment of thyroid nodules and is currently endorsed by several groups worldwide (Bartolazzi *et al*, 2001; Cerutti *et al*, 2004; Jacques *et al*, 2005; Takano *et al*, 2005; Weber *et al*, 2005; Eszlinger *et al*, 2007).

Very recently our study group has evaluated a set of 10 genes (Cerutti *et al*, 2004; Wang *et al*, 2004; Fuhrer *et al*, 2005; Takano *et al*, 2005; Weber *et al*, 2005; Eszlinger *et al*, 2007), identified through transcriptome analysis, as discriminative markers in thyroid nodules from patients living in a region with borderline iodine deficiency. In this study, we confirmed the applicability of six-marker genes (*ADM3/HGD1/LGALS3/PLAB/TFF3/TG*) for the distinction of benign and malignant thyroid tumours including follicular thyroid neoplasia with a sensitivity of 91%, a specificity of 100%, a positive predictive value (PPV) of 1.0 and a negative predictive value (NPV) of 0.94 (Krause *et al*, 2008).

In the present study, we investigate the possibility that these markers can be applied for the differential diagnosis in FNAB. To this aim, an independent, prospective series of 156 FNABs from patients undergoing thyroid surgery for a cold thyroid nodule was analysed and results of molecular diagnostics were compared with the definite histological outcome comprising 18 thyroid cancers (7 follicular thyroid cancers and 11 papillary thyroid cancers), 93 benign thyroid tumours (40 follicular adenoma (FA) and 53 adenomatous nodules) and 45 surrounding normal thyroid tissues.

## Materials and methods

### Thyroid samples

In all, 111 patients undergoing surgery for a thyroid tumour were studied. Fine-needle aspirates of the thyroid nodules were obtained prospectively (i) *in vivo* in a private practice for endocrinology and nuclear medicine in Lüneburg (*n*=66) and (ii) *ex vivo*, immediately after surgical removal of the thyroid tumour in the Department of Surgery in Schkeuditz and the University Hospital Halle (*n*=45). Fine-needle aspirates were gained for cytological and molecular investigation. Cytological investigation was performed by a local pathologist in the cohort of *in vivo* FNAB and by a pathologist from our University Hospital in the cohort of *ex vivo* FNAB. Fine-needle aspiration biopsies were classified as recommended by the NCI Thyroid Fine-needle Aspiration (FNA) State of the Science Conference in 2007 (Baloch *et al*, 2008). Thyroid tumours were classified according to the latest WHO classification of tumours of endocrine organs (DeLellis *et al*, 2004). Cytological and histological results of the investigated thyroid carcinomas are summarised in Table 1. In total, FNABs were obtained from 93 solitary benign cold thyroid nodules comprising 53 adenomatous nodules and 40 FA, 7 follicular thyroid carcinomas (FTC) and 11 papillary thyroid carcinomas (PTC), including 3 follicular variant PTC (FVPTC). In addition, FNABs were performed from surrounding normal thyroid tissues in the *ex vivo* series. Patients did not receive thyroid medication and were euthyroid at the time of surgery (a list of clinical data and TNM stage of the thyroid cancers can be provided upon request). Informed consent was obtained from all patients. The local ethics committee approved the study.

### RNA extraction and quantitative real-time PCR

Needle remnants were washed out and transferred into TRIzol reagent (Invitrogen, Carlsbad, CA, USA). RNA extraction and cDNA synthesis were carried out as previously described (Eszlinger *et al*, 2005; Fuhrer *et al*, 2005) and expression of house-keeping gene *β*-actin (*ACTB*) was demonstrated in all samples by RT–PCR. Real-time PCR (LightCycler System, LightCycler–DNA Master SYBR Green I Kit; Roche, Mannheim, Germany) was performed using intron spanning primers for *ADM3* (Cerutti *et al*, 2004), *HGD1* (Eszlinger *et al*, 2007), *LGALS3* (Takano *et al*, 2005), *PLAB* (Weber *et al*, 2005), *TFF3* (Takano *et al*, 2004, 2005), *TG* and the house-keeping gene *β*-actin (*ACTB*) as previously described (Krause *et al*, 2008). For each PCR, annealing temperatures and MgCl_2_ concentrations were optimised to create a one-peak-melting curve (primer sequences and PCR conditions are available upon request).

### Statistical analysis

The fold difference (*n*) in upregulation or downregulation of mRNA expression was calculated as follows:



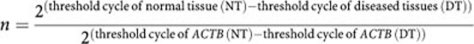



‘Normal tissue’ corresponds to the surrounding thyroid tissue of tumours assembled in the *ex vivo* series. For the molecular discrimination of benign and malignant thyroid tumours, mRNA expression levels in 40 FAs and 53 adenomatous nodules were compared with mRNA expression levels in 18 thyroid cancers (7 FTC and 11 PTC). For the molecular discrimination of follicular thyroid tumours, mRNA expression levels in 40 FAs were compared with mRNA expression levels in 10 malignant follicular thyroid neoplasias (including three FVPTC). The Mann–Whitney *U*-test within the SPPS software (version 11.5; SPSS, Chicago, IL, USA) was used for statistical analysis of mRNA expression differences and a *P*-value of <0.05 was defined as statistically significant.

In case of significant expression, differences between benign *vs* malignant thyroid tumours and between FA *vs* malignant follicular thyroid neoplasia, cross-validation analysis was performed to calculate the optimal discriminative cutoff value between both entities using SPSS software (version 11.5; SPSS). Additionally, specificity and sensitivity as well as PPV and NPV and accuracy were calculated for each of these marker genes and marker gene combinations.

## Results

Presence of *β*-actin (*ACTB*) and thyroglobulin (*TG*) mRNA expression was confirmed in all FNAB samples and was mandatory for further marker gene analysis. Messenger RNA expression for individual marker genes was demonstrated in 109 (*TFF3*), 99 (*LGALS3*), 95 (*ADM3*), 84 (*HGD1*) and 67 (*PLAB*) of 111 fine-needle biopsies obtained from the thyroid tumours and in all 45 samples obtained from normal thyroid tissues. There was no difference in mRNA retrieval or LightCycler PCR (threshold cycles) quantification between fine-needle biopsies obtained *in vivo* or *ex vivo*.

On a single gene basis, FNAB of histologically proven malignant thyroid tumours (*n*=18) showed significantly lower mRNA expression levels for *TFF3* and *HGD1* and significantly higher mRNA levels for *ADM3* and *LGALS3* compared with FNAB of benign thyroid tumours (*n*=93) (Table 2A).

**Figure 1:**
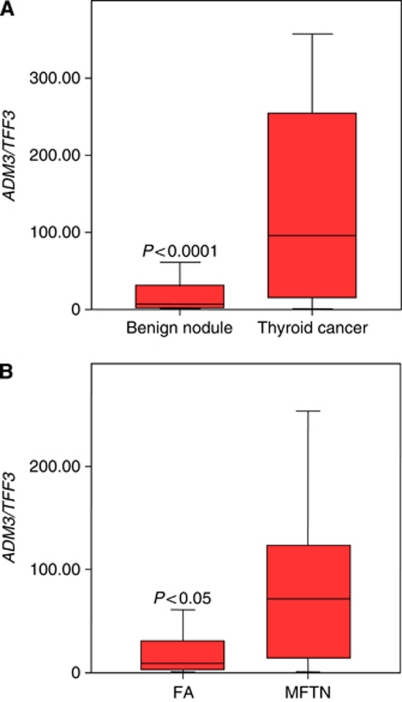
Molecular FNAB analysis in benign and malignant thyroid tumours. Box plots showing median and distribution (box area=50% of samples) of the *ADM3/TFF3* marker gene combination, which allowed statistically significant separation (*P*<0.0001 (**A**) and *P*<0.05 (**B**); MWU-test) in (**A**) FNAB of benign thyroid nodules (*n*=93) and thyroid cancers (*n*=18); (**B**) FNAB of FA (*n*=40) and malignant follicular thyroid neoplasia (MFTN; *n*=10 including seven FTC and three follicular variant PTC). Ratios were calculated as described in Materials and Methods.

**Figure 2:**
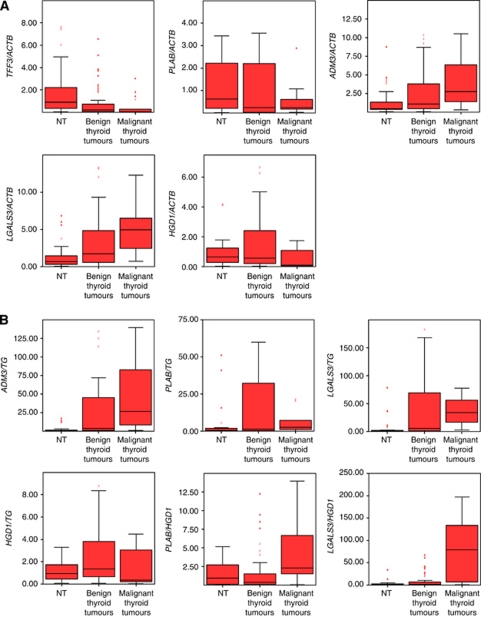

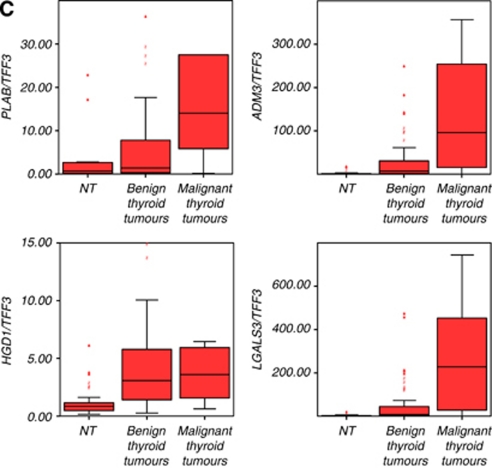
Molecular FNAB analysis in benign and malignant thyroid tumours and NT. Box plots showing median and distribution (box area=50% of samples) of all investigated marker genes and marker gene combinations. (**A**) *TFF3/ACTB*, *PLAB/ACTB*, *ADM3/ACTB*, *LGALS3/ACTB*, *HGD1/ACTB* (**B**) *ADM3/TG*, *PLAB/TG*, *LGALS3/TG*, *HGD1/TG*, *PLAB/HGD1*, *LGALS3/HGD1* (**C**) *PLAB/TFF3*, *ADM3/TFF3*, *LGALS3/TFF3*, *HGD1/TFF3*. Data of statistical analyses (benign *vs* malignant) are expressed in Table 2A.

When calculating two-gene expression ratios, 5 out of 7 previously described marker gene sets (Krause *et al*, 2008) showed significant expression differences in FNAB of thyroid cancers *vs* benign thyroid tumours, with the remainder showing the same trend for upregulation or downregulation as in our previous study (Table 2A; Figure 1A). In addition, three novel gene combinations were found to be significantly altered in benign *vs* malignant thyroid tumours (*ADM3/TG*, *HGD1/TG* and *LGALS3/TG*). There were no significant differences between benign thyroid nodules and normal thyroid tissues expression in all investigated genes and gene combinations (Figure 2).

After determination of discriminative cutoff values as described in the Materials and Methods section, diagnostic usefulness was calculated and showed a specificity of up to 87% and a sensitivity of up to 82% for the distinction of benign and malignant thyroid tumours (NPV up to 0.78, PPV up to 0.84; accuracy 0.78) (Table 2A).

No improvement in diagnostic accuracy was found when calculating the product from best discriminating gene ratios *LGALS3/HGD1* x *PLAB/TFF3* or *LGALS3/HGD1* x *LGALS3/TG*.

A specific molecular discrimination of FNAB from malignant follicular thyroid neoplasia *vs* FA was possible by quantification of *ADM3* expression, and by application of two *ADM3*-based gene combinations (*ADM3/TFF3*, *ADM3/TG*) (Figure 1B). Furthermore, a high significant discriminative result was obtained by calculating the product of *ADM3/ACTB* x *ADM3/TFF3* expression, which clearly outperformed the solitary use of *ADM3*-based gene sets (Table 2B). On the basis of the calculated discriminative cutoff values, overall specificity and sensitivity was up to 87% and 90%, respectively (NPV up to 0.94, PPV up to 0.86; accuracy 0.84) (Table 2B). Furthermore, we observed the same trend for upregulation or downregulation in all described marker gene sets of our previous study (Table 2B).

A direct comparison of FNAB results from all proven thyroid malignancies with the best discriminative marker gene sets (*LGALS3/HGD1*, *LGALS3/TG*, *ADM3/ACTB*, *ADM3/TFF3*, *ADM3/TG*, *ADM3/ACTB* x *ADM3/TFF3*) revealed correct detection of a malignant follicular thyroid neoplasia in 8 out of 9 FNAB classified as ‘follicular neoplasm (FN)/suspicious for FN’. One PTC with a non-diagnostic FNAB was correctly identified by the proposed marker set. In three cases of PTC with benign FNAB, one case was correctly classified as malignant by the proposed marker set, one case was misclassified as false negative and in one case the proposed markers were not detected in the needle remnant (Table 1).

Furthermore, we analysed *ADM3*-based marker gene expression in nine cases with ‘FN/suspicious for FN’ – FNAB result, which were available in the group of histological proven benign nodules and compared them with *ADM3*-based marker gene expression in nine cases with ‘FN/suspicious for FN’ – FNAB result in the group of histological proven thyroid cancer. This small subanalysis showed *ADM3/TFF3* as the best predictor of benignancy (specificity 89%) and *ADM3/ACTB* as the best predictor of malignancy (sensitivity 89%) with only one misclassified case per group.

## Discussion

In a previous pilot study we demonstrated that six recently identified marker genes (*ADM3/HGD1/LGALS3/PLAB/TFF3/TG*) can be successfully applied for a molecular discrimination of benign and malignant thyroid tumours including follicular thyroid neoplasia (Krause *et al*, 2008) using surgically removed tissue samples. We now extend this study to the analysis of FNAB of thyroid tumours. To this aim, a prospective, independent FNAB series was collected and results of molecular FNAB diagnosis were compared with the histological outcome of 111 surgically removed thyroid tumours.

The inclusion of FVPTC together with FTC in the molecular diagnostic group of malignant follicular thyroid neoplasia (as already performed in our previous pilot study) can be justified since FNAB from FVPTC evoke the same cytopathological difficulties and uncertainties as FNAB from FA and FTC (Hamberger *et al*, 1982). Furthermore, FVPTC share important molecular fingerprints with FTC rather than with PTC, for example, concerning the prevalence of RAS- *vs* BRAF-mutations (Nikiforov, 2011).

In this series, we largely confirm our previous results. Thus, we found statistically significant expression differences in 5 out of 7 previously proposed two-marker gene combinations and demonstrate their usefulness for the molecular discrimination of FNAB from benign and malignant thyroid tumours. Using a combination of at least two-marker gene sets, a specificity of up to 87% (*LGALS3/HGD1*) and a sensitivity of up to 82% (*LGALS3/TG*) emphasises that molecular analysis of FNAB is a promising tool to improve the differential diagnosis of thyroid nodules (Hegedüs, 2010). For the subgroup of follicular thyroid neoplasia, the identification of molecular markers that separate FNAB from FA and carcinoma was more complex. Statistical significance was only found for two ADM3-based marker gene combinations; however, the same trend of upregulation or downregulation was observed for all others previously published marker gene ratios (Krause *et al*, 2008). The product calculation of *ADM3/ACTB* x *ADM3/TFF3* provided a two-gene marker tool with a high NPV (0.94) and diagnostic accuracy (0.84) for the exclusion of follicular thyroid malignancy.

In our view, these findings are remarkable for two reasons: first, they re-emphasise the diagnostic potential of individual marker genes, which have now been replicated in an independent thyroid tumour series. Second, and importantly, these marker genes are of diagnostic value in FNAB, despite a much lower yield of genetic material for molecular analysis and are therefore ‘proof of principle’.

The lower yield in the needle wash-out of thyroid tumour FNAB could be one explanation, why most but not all of the previously identified marker gene sets were statistically significant in FNABs of benign and malignant thyroid tumours and possibly also accounts for the fact that identification of discriminative marker gene combinations in FNAB of follicular neoplasia was limited. In addition, the number of FNAB with histologically proven malignant follicular thyroid neoplasia (including follicular thyroid cancer and follicular variant papillary thyroid cancer) was much lower in this prospective study than in our previous investigation.

Recent other studies did also address *TFF3* mRNA as a promising differentiation marker in FNAB of thyroid neoplasia (Takano *et al*, 2005; Ducena *et al*, 2011; Patel *et al*, 2011). Our results confirm their findings of a lower *TFF3* mRNA expression in malignant *vs* benign thyroid tumours as well as in malignant follicular thyroid neoplasia *vs* FA. In the latter, we failed to show statistical significance, which might be a problem of statistical power due to the low number of FTC and FVPTC cases. However, the hitherto non-described ratio of *ADM3/TFF3* and the product of *ADM3/ACTB* x *ADM3/TFF3* represent a potential tool for discrimination of benign and malignant follicular thyroid neoplasia.

An important advantage of TFF3 mRNA analysis is that it can serve as an appropriate internal control mRNA, since its expression is restricted to thyroid epithelial cells and cannot be found in blood cells (Takano *et al*, 1998), which are contaminating more or less every FNAB sample. Additionally, we assume that the same applies for *ADM3*, since we have not found any hints of *ADM3* mRNA expression in human blood cells in a thorough literature research. Interestingly, even in case of high amounts of benign background (up to 80% dilution), the classifier used by Chudova *et al* (2010) correctly recognised thyroid malignancy in the majority of their investigated FNA samples.

To our knowledge, these are the first data on *ADM3*-based marker gene expression analyses in a prospective thyroid FNAB series, comparing molecular markers with the definite histological outcome. The present results and the recent exciting data on the use of high-dimensionality genomic data, mutation analysis and miRNA expression analysis in FNAB reported by other investigators (Cheung *et al*, 2001; Salvatore *et al*, 2004; Lubitz *et al*, 2006; Pizzolanti *et al*, 2007; Sapio *et al*, 2007; Nikiforova *et al*, 2008; Nikiforov *et al*, 2009; Chudova *et al*, 2010) justify an optimistic view on the potential of molecular FNAB diagnosis.

Based on our present results, we would like to discuss the following diagnostic algorithm to guide the patient to surgery or not:

It stands out of question that any thyroid nodule with typical clinical findings highly suspicious for thyroid malignancy (hard/fixed thyroid lumb, hoarseness, enlarged lymph node(s)) and/or patients with accompanying risk factors (e.g., radiation in childhood, positive family history) should be generously referred to surgery after FNAB. Additionally, thyroid nodules presenting with ultrasound criteria, for example, based on the TIRADS classification (Horvath *et al*, 2009) with high sensitivity for the detection of thyroid malignancy (TIRADS IVB–VI) should also be referred to surgery after FNAB. However, the vast majority of thyroid nodules presents with ultrasound criteria typical of a benign thyroid lesion (TIRADS II–IVA). However, even in the hand of an experienced US investigator, thyroid nodules with TIRADS III and IVA are implying a remaining rest risk for thyroid malignancy of <5–10% and should therefore considered for FNAB (Horvath *et al*, 2009). If FNAB in such cases results in (based on NCI Thyroid FNA; Baloch *et al*, 2008) (i) repeatedly non-diagnostic, (ii) a follicular lesion of undetermined significance, (iii) a FN or (iv) a Hurthle cell neoplasm, we would see placed our two-gene markers *LGALS3/HGD1 and LGALS3/TG* as well as *ADM3/ACTB* x *ADM3/TFF3* as helpful tools to decide either for watchful waiting or for surgery.

However, the implementation and validation of such algorithms can only be achieved by future joint research efforts of multiple thyroid centres with the conviction that molecular FNAB diagnosis should become a supplementary tool in future management of thyroid nodular disease, which could ultimately save patients from unnecessary thyroid surgery and favourably impact thyroid disease-related health care costs.

## Figures and Tables

**Table 1:**
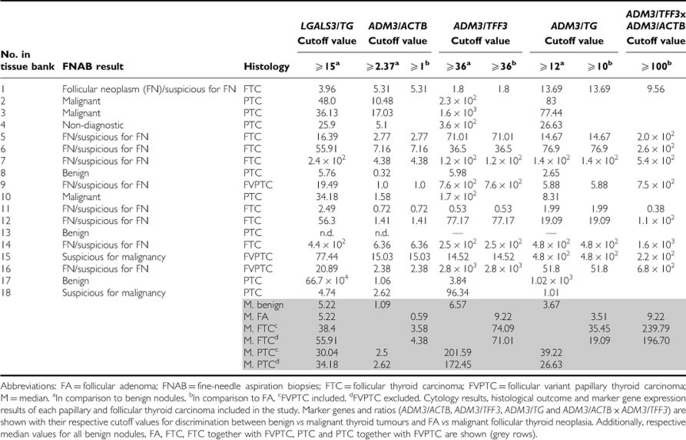
Comparison of cytological and histological outcome in thyroid cancers with marker gene expression in the FNAB

**Table 2A:**
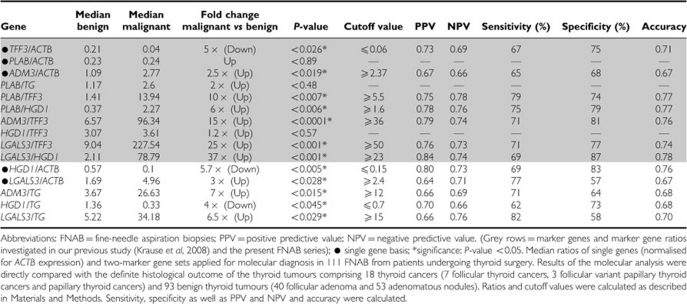
Median ratios of five single genes and two-marker gene sets applied for molecular diagnosis in FNAB of benign (*n*=93) and malignant (*n*=18) thyroid tumours

**Table 2B:**
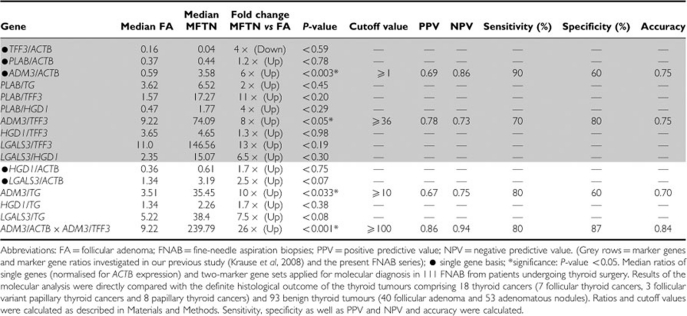
Median ratios of five single genes and two-marker gene sets applied for molecular diagnosis in FNAB of benign (*n*=40) and malignant (*n*=10) follicular thyroid neoplasia (MFTN)
